# Agonist efficacy at the β_2_AR is driven by the faster association rate of the G_s_ protein

**DOI:** 10.3389/fphar.2025.1367991

**Published:** 2025-03-27

**Authors:** Clare R. Harwood, David A. Sykes, Theo Redfern-Nichols, Owen Underwood, Colin Nicholson, Armin N. Khoshgrudi, Eline J. Koers, Graham Ladds, Stephen J. Briddon, Dmitry B. Veprintsev

**Affiliations:** ^1^ Division of Physiology, Pharmacology and Neuroscience, School of Life Sciences, University of Nottingham, Nottingham, United Kingdom; ^2^ Centre of Membrane Proteins and Receptors (COMPARE), University of Birmingham and University of Nottingham, Nottingham, United Kingdom; ^3^ Z7 Biotech Ltd., Nottingham, United Kingdom; ^4^ Department of Pharmacology, University of Cambridge, Cambridge, United Kingdom

**Keywords:** G protein-coupled receptor, β2-adrenoceptor, efficacy, kinetics, association rate kon, dissociation rate koff

## Abstract

**Introduction:**

The β_2_-adrenoceptor (β_2_AR) is a class A G protein-coupled receptor (GPCR). It is therapeutically relevant in asthma and chronic obstructive pulmonary disease (COPD), where β_2_AR agonists relieve bronchoconstriction. The β_2_AR is a prototypical GPCR for structural and biophysical studies. However, the molecular basis of agonist efficacy at the β_2_AR is not understood. We hypothesised that the kinetics of GPCR–G protein interactions could play a role in determining ligand efficacy. By studying a range of agonists with varying efficacy, we examined the relationship between ligand-induced mini-G_s_ binding to the β_2_AR and ligand efficacy, along with the ability of individual ligands to activate the G protein in cells.

**Methods:**

We used NanoBRET technology to measure ligand-induced binding of purified Venus-mini-G_s_ to β_2_AR-nLuc in membrane preparations under both equilibrium and kinetic conditions. In addition, we examined the ability of these β_2_AR agonists to activate the heterotrimeric G_s_ protein, measured using the G_s_-CASE protein biosensor in living cells. This assay detects a reduction in NanoBRET between the nano-luciferase (nLuc) donor on the Gα subunit and Venus acceptor on the Gγ upon G_s_ protein activation.

**Results:**

The 12 β_2_AR agonists under study revealed a broad range of ligand potency and efficacy values in the cellular G_s_-CASE assays. Kinetic characterisation of mini-G_s_ binding to the agonist β_2_AR complex revealed a strong correlation between ligand efficacy values (E_max_) and mini-G_s_ affinity (*K*_d_) and its association rate (*k*_on_). In contrast, there was no correlation between ligand efficacy and reported ligand dissociation rates (or residence times).

**Conclusion:**

The association rate (*k*_on_) of the G protein to the agonist β_2_AR complex is directly correlated with ligand efficacy. These data support a model in which higher-efficacy agonists induce the β_2_AR to adopt a conformation that is more likely to recruit G protein. Conversely, these data did not support the role of agonist binding kinetics in determining the molecular basis of efficacy.

## Introduction

G protein-coupled receptors (GPCRs) are the largest family of membrane proteins in the human genome and are responsible for modulating a broad range of hormonal, neurological, and immune responses. GPCR-directed therapeutics currently target over 100 diverse receptors and represent 34% of all US Food and Drug Administration (FDA)-approved drugs, making them the most widely targeted receptors (Hauser et al., 2017). Despite their therapeutic importance, the molecular basis of ligand efficacy—the ability of a drug to affect GPCR signal transduction—is not fully understood. It is hoped that a deeper understanding of the molecular basis of efficacy will aid in more rational drug design.

The process of GPCR activation involves agonist binding, a ligand-induced conformational change in the receptor and the subsequent recruitment and activation of a G protein. Several studies have implicated ligand residence time in the molecular basis of efficacy at GPCRs. For example, a positive correlation has been observed between the efficacy of seven agonists at the muscarinic M3 receptor and 10 agonists at the adenosine A_2A_ receptor (A_2A_R) with their ligand residence time (Sykes et al., 2009b; Guo et al., 2012). Conversely, no correlation between efficacy and residency time was found for ligands at the adenosine A_1_ receptor (Louvel et al., 2014).

Biophysical studies have shown that agonists shift the receptor conformational landscape in favour of a unique active conformation, compared to the unliganded state (Deupi and Kobilka, 2010; Mary et al., 2012; Nygaard et al., 2013), but how conformational differences in a population translate to greater or lesser signalling responses remains to be fully elucidated. Structural studies have found little differences in GPCR conformations adopted by ligand-bound GPCR–G-protein complexes (Masureel et al., 2018; Zhang et al., 2020). However, using nuclear magnetic resonance (NMR), Liu et al. (2012) showed efficacy-dependent differences in the conformational state of β_2_AR bound to different agonists prior to G protein binding. Similar results have been observed for the β_1_AR (Grahl et al., 2020; Jones et al., 2024) and A_2A_R (Ye et al., 2016). Alternatively, some studies (Nikolaev et al., 2006; Gregorio et al., 2017) show correlations between ligand efficacy and the rate of GPCR and G protein activation, suggesting a key role for G protein binding kinetics in dictating pharmacological efficacy.

Consequentially, we aimed to delineate the roles of ligand binding and receptor–G protein binding kinetics in agonist efficacy. We focused on the β_2_-adrenoceptor (β_2_AR), a prototypical class A GPCR, which is one of the most structurally, functionally, and therapeutically well-characterised GPCRs. The β_2_AR is also an essential target in the treatment of asthma and COPD, and as a result, a wide range of clinically used agonists of varying efficacies have been developed to target the β_2_AR, which could be utilised in this study.

G proteins are heterotrimeric, consisting of α, β, and γ subunits The Gα subunit comprises of a helical and GTPase domain. Full-length heterotrimeric G proteins are dynamic complexes that are difficult to isolate. To overcome this, we chose to utilise mini-G proteins (Carpenter and Tate, 2016) as tools to study the dynamics of β_2_AR activation. The mini-G_s_ protein is the isolated GTPase domain of the Gα subunit, which has been engineered with several thermostabilising mutations that make it a rigid protein, locked in its active state, as shown in the agonist-bound A_2A_R-mini-G_s_ structure (Carpenter et al., 2016; Carpenter and Tate, 2017). These mini-G proteins have also been converted into convenient probes that report the active state of a GPCR (Wan et al., 2018).

We investigated the binding kinetics and affinity of fluorescently labelled (Venus-fused) mini-G_s_ proteins for the β_2_AR in complex with a set of agonists of varying efficacy, from partial to full agonists. In addition, we correlated ligand binding affinities, residence times, and efficacy at the level of heterotrimeric G_s_ protein activation for these agonists.

## Materials, instruments and software

### Materials

The T-REx™-293 Cell Line was obtained from Invitrogen (CA, United States). T75 and T175 mammalian cell culture flasks were purchased from Fisher Scientific (Loughborough, United Kingdom). All cell culture reagents, including Hank’s balanced salt solution (HBSS), phosphate-buffered saline (PBS), and foetal calf serum (FCS), were purchased from Sigma Aldrich (Gillingham, United Kingdom), except for blasticidin, which was obtained from Gibco™ (MA, United States), and Zeocin™. Polyethylenimine (PEI) (25 kDa) was obtained from Polysciences Inc. (PA, United States), and the culture plates were obtained from Greiner Bio-One (code 655098 Kremsmünster, Austria).

HisTrap FF crude 5-mL columns were obtained from GE Healthcare (IL, United States). Vivaspin protein concentrators were obtained from Sartorius (Göttingen, Germany). Slide-A-Lyzer Dialysis Cassettes, NuPAGE LDS Sample Buffer, NuPAGE 4%–12% Bis-Tris 15 × 1.0 mm well gels, NuPAGE MOPS SDS Running Buffer, PageRuler Prestained Protein Ladder, were all obtained from Thermo Fisher (MA, United States).

Salmeterol was obtained from Tocris (Bristol, U.K). Formoterol hemifumarate was obtained from APExBIO (TX, United States), and BI-167-107 was obtained from Boehringer Ingelheim (Ingelheim, Germany). Compound 26 was a gift from Novartis. (±)-Epinephrine hydrochloride, noradrenaline, salbutamol hemisulfate, and isoprenaline hydrochloride were purchased from Sigma-Aldrich (Gillingham, United Kingdom). Dobutamine hydrochloride was obtained from Merck Life Sciences, UK. Isoxsuprine hydrochloride, ritodrine hydrochloride, and tulobuterol were obtained from CliniSciences Limited. Nano-Glo luciferase substrate was obtained from Promega (WI, United States). All other chemicals were purchased from Sigma-Aldrich (Gillingham, United Kingdom).

### Instruments and software

BMG PHERAstar FSX plate reader (BMG Labtech, Offenburg, Germany), fitted with BRET1 plus optic module (ex. 475/30 nm, em. 535/30 nm) and MARS software, was purchased from BMG Labtech (Offenburg, Germany). GraphPad Prism 9 was purchased from GraphPad Software (San Diego, United States). Microsoft Excel™ XP was purchased from Microsoft (Washington, United States).

## Methods

### Molecular biology

The construct pcDNA4TO-TwinStrep (TS)-SNAP-β_2_AR was generated through the amplification of the SNAP and β_2_AR sequences from the pSNAPf-ADRB2 plasmid (NEB) and inserted into pcDNA4TO-TS using Gibson assembly (Heydenreich et al., 2017). pcDNA4TO-TS-SNAP-β_2_AR-nLuc was generated by Dr. Brad Hoare through the amplification of pcDNA4TO-TS-SNAP-β_2_AR and nanoLuc, with the insertion of nanoLuc into pcDNA4TO-TS-SNAP-β_2_AR via Gibson assembly. Both constructs used a signal peptide based on the 5HT_3A_ receptor to increase protein folding and expression. The CASE G_s_ (or G_s_-CASE) protein constructs were designed and optimised by the Schulte Lab (Schihada et al., 2021) and were obtained from Addgene. Mammalian Venus-fused mini-G_s_ constructs were a kind gift from Nevin Lambert (Wan et al., 2018). For the bacterial expression of Venus-mini-G_s_ and mini-G_s_, protein encoding DNA sequences were amplified from the corresponding mammalian constructs and inserted into the pJ411 vector containing MKK-HIS10-TEV N-terminal tag (Sun et al., 2015) via Gibson assembly, yielding the constructs MKK-HIS10-TEV-mini-G_s_ and MKK-HIS10-TEV-Venus-mini-G_s_.

### Transfection and mammalian cell culture

pcDNA4TO-TS-SNAP-β_2_AR or pcDNA4TO-TS-SNAP-β_2_AR-nLuc was stably transfected into T-REx™-293 cells (Invitrogen) using PEI. A stable mixed population was selected by resistance to 5 μg/mL blasticidin and 20 μg/mL zeocin. Stable cell lines were maintained in high-glucose DMEM (Sigma D6429) with 10% FBS, 5 μg/μL blasticidin, and 20 μg/μL zeocin at 37°C in a humidified atmosphere of 5% CO_2._ When ∼70% confluent, TS-SNAP-β_2_AR or TS-SNAP-β_2_AR-nLuc expression was induced with 1 μg/mL tetracycline. Cells were left to express for 50 h before harvesting for assays. The T-REx™-293 pcDNA4TO-TS-SNAP-β_2_AR-CASE G_s_ stable cell line was generated by stably transfecting the CASE G_s_ constructs into the T-REx™-293 pcDNA4TO-TS-SNAP-β_2_AR using PEI. A mixed population stable cell line was generated by selection with 500 μg/mL G418, and then a single colony population was generated via FACS.

### Membrane preparations of TS-SNAP-β_2_AR-nLuc

For membrane preparation, all steps were conducted at 4°C to avoid tissue degradation. Cell pellets were thawed and re-suspended using ice-cold buffer containing 10 mM HEPES and 10 mM EDTA (pH 7.4). The suspension was homogenised using an electrical homogeniser (ULTRA-TURRAX, IKA-Werke GmbH, Germany) and subsequently centrifuged at 1,200 × g for 5 min. The pellet obtained, containing cell nucleus and other heavy organelles, was discarded, and the supernatant was centrifuged for 30 min at 48,000 × g at 4°C (Beckman Avanti J-251 Ultra-centrifuge; Beckman Coulter). The supernatant was discarded, and the pellet was re-suspended in the same buffer (10 mM HEPES and 10 mM EDTA; pH 7.4) and centrifuged again for 30 min as described above. Finally, the supernatant was discarded, and the pellet was re-suspended in ice-cold 10 mM HEPES and 0.1 mM EDTA (pH 7.4). Protein concentration determination was carried out using the bicinchoninic acid assay kit (Sigma-Aldrich) with BSA as the standard. The final membrane suspension was aliquoted and maintained at −80°C until required for the assays.

### Solubilisation of the TS-SNAP-β_2_AR or TS-SNAP-β_2_AR-nLuc

TS-SNAP-β_2_AR or TS-SNAP-β_2_AR-nLuc was solubilised from stably transfected T-REx^TM^-293 cell membranes, as described previously (Harwood et al., 2024). Solubilisation was carried out using 1% DDM (w/v) in 20 mM HEPES, 5% (v/v) glycerol, and 150 mM NaCl, pH 8, at 4°C for 2–3 h. Samples were clarified by ultracentrifugation at 4°C for 1 h at 100,000 × g.

### Production of mini-G_s_

His-TEV-Venus-mini-G_s_ and His-TEV-mini-G_s_ were expressed in NiCo21(DE3) *E.* coli, cultured in Terrific Broth (Gibco). 1L cultures were induced with 1 mM isopropyl β-D-1-thiogalactopyranoside (IPTG) at OD = 0.6 and incubated for a further 20 h at 20°C and 225 RPM. Pellets from 1L cultures were thawed on ice, and re-suspended in 50 mL lysis buffer (20 mM HEPES, pH 7.5, 500 mM NaCl, 40 mM imidazole, 10% glycerol, 8 mM β-mercaptoethanol (BME), 1 μM guanosine diphosphate (GDP), complete protease inhibitors (Roche), DNase I, and lysozyme) using a Dounce homogeniser. Lysis occurred on ice via sonication, using a Vibra-Cell probe sonicator with 5 × 10-s pulses, 30 s apart. The lysate was loaded onto the HisTrap FF crude 5-mL column, using ÄKTA™ start protein purification system at a flow rate of 5 mL/min. The system and column had been equilibrated with 10 column volumes (CV) of buffer A (20 mM HEPES, 500 mM NaCl, 40 mM imidazole, 10% glycerol, 8 mM BME, and 1 μM GDP). Unbound protein was washed out with 10 CV of buffer A. Bound protein was then eluted over an 8 CV gradient of 0% to 100% buffer B at a flow rate of 5 mL/min (Buffer B = 20 mM HEPES, 500 mM NaCl, 400 mM imidazole, 10% glycerol, 8 mM BME, and 1 μM GDP). The presence of His-TEV-Venus-mini-G_s_ and His-TEV-mini-G_s_ was confirmed by SDS-PAGE analysis and InstantBlue staining for protein. Pooled elution fractions were then concentrated using 10,000 or 30,000 molecular weight cutoff (MWCO) Vivaspin protein concentrators by centrifugation at 3000 *×* g and 4°C for 15-min intervals over 2–3 h. Protein was exchanged into assay buffer using Slide-A-Lyzer 10,000 or 30,000 MWCO dialysis cassettes for untagged and Venus-tagged mini-G_s_ protein samples, respectively. Dialysis occurred overnight at 4°C under constant stirring. The assay buffer consisted of 20 mM HEPES, 150 mM NaCl, 10% glycerol, 8 mM BME, and 1 μM GDP. The purified mini-G_s_ protein was flash-frozen using liquid nitrogen and stored at −80°C.

### Membrane-based TS-SNAP-β_2_AR-Venus-mini-G_s_ NanoBRET binding assays

The assay buffer, consisting of HBSS (Sigma H8264) containing 10 mM HEPES, 0.1% BSA, and 0.1% ascorbic acid, pH 7.4, was used in all NanoBRET assays. For recruitment assays, varying concentrations of β_2_AR agonists were used to recruit Venus-mini-G_s_ to the TS-SNAP-β_2_AR. Assays were run in 50 μL volumes in white 384-well OptiPlate (Revvity). Receptor, ligand, 0.3 μM mini-G_s_ proteins, and 10 μM furimazine were added to the plate and incubated for 60 min at room temperature before reading on PHERAstar FSX using the BRET1 module. For kinetic assays, in which the affinity of Venus-mini-G_s_ for the agonist-bound TS-SNAP-β_2_AR-nLuc receptors was measured over time, assays were run in 50 μL volumes in white 384-well OptiPlate. Varying concentrations (10–300 nM) of Venus-mini-G_s_ were added to assay plates. TS-SNAP-β_2_AR membranes were pre-incubated with saturating concentrations (100x EC_50_) of selected β_2_AR agonists and furimazine for 15 min prior to addition to the plate. TS-SNAP-β_2_AR membranes were added to the plate offline and mixed with the Venus-mini-G_s_ on a plate shaker (MixMate, Eppendorf) at 600 RPM for 10 s. The mixture was then immediately read on PHERAstar FSX as described above, with readings taken over a period of 240 min.

### G_s_-CASE activation assays

For G_s_-CASE activation assays, a single population of T-REx™-293 stably expressing pcDNA4TO-TS-SNAP-β_2_AR and CASE G_s_ was plated at 50,000 cells/well in 96-well plates, in a volume of 100 μL, and induced for 48 h with 1 μg/mL tetracycline at 37°C and 5% CO_2_. Plates were washed once with 100 μL/well assay buffer (HBSS containing 10 mM HEPES, 0.1% BSA, and 0.1% ascorbic acid) prior to the addition of 90 μL/well of assay buffer containing 10 μM furimazine, diluted in assay buffer, to achieve a final concentration of 8 μM. The plates were incubated at 37°C and 5% CO_2_ for 20 min. A white back seal was placed on the underside of the plate, and luminescence was read on a PHERAstar FSX using the BRET1 module for 3 min to establish a baseline BRET signal. The plate reader was then paused, and 10 μL of ×10 ligand dilutions were added accordingly. Readings were taken over a period of 30 min.

### Mathematical modelling

The previously described ordinary differential model (ODE) of the cubic ternary complex model (Weiss et al., 1996), with additional reactions to simulate the G protein activation cycle, was used (Woodroffe et al., 2009; Bridge et al., 2018). The model, encoded in COPASI (Hoops et al., 2006), includes ligand binding, receptor activation, G protein binding, and the G protein cycle, whereby the model output is activated G protein Gα_GTP_ and receptor occupancy (Bridge et al., 2018). Prior to the addition of the ligand, we first compute the system for 10^6^ s. To enable the simulation of the data, the cooperativity factor β (see Supplementary Figure 7; Supplementary Table 3) was varied, and simulations were performed. Steady state was reached after 5 min, and outputs are shown after 10 min.

### Data analysis

All non-linear regression and statistical analyses were performed using GraphPad Prism 9. Multiple replicates were combined, such as TR-FRET equilibrium binding curves and mini-G_s_ equilibrium recruitment curves, as shown in Supplementary Material. Data points for each replicate were normalised to the maximum value obtained for each ligand in each experiment. Competition ligand-binding data were fitted to a one-site model (Equation 1).

$$Y=\frac{Bottom+\left(Top-Bottom\right)}{\left(1+10^{\left(x-{LogIC}_{50\;}\right)}\right)},$$ (1)

where Y is the binding of tracer, x = Log [ligand], IC_50_ is the concentration of the competing ligand that displaces 50% of radioligand-specific binding.

CASE G_s_ activation data from individual experiments were fitted to sigmoidal (variable slope) curves using a “four-parameter logistic equation” (Equation 2):

$$Y=Bottom+\frac{\left(Top-Bottom\right)}{1+10^{\left(logEC50-X\right)*Hillslope}},$$ (2)

where Bottom is the plateaus of the agonist concentration response curve and Top is the basal response (fixed to 1). LogEC_50_ is the concentration of the agonist that produces a half-maximal effect, and the Hillslope is the unitless slope factor or Hillslope, which was fixed to −1.

Mini-G_s_ association data were fitted to a global fitting model (Equation 3) using GraphPad Prism 9.2 to simultaneously calculate *k*_on_ and *k*_off_ using the following equations, where *k*_obs_ equals the observed rate of association and L is the concentration of mini-G_s_.

$$K_{d}=\frac{k_{\text{off}}}{k_{\text{on}}},$$

L=Hotnm*1e−9,

$$K_{\text{ob}}=k_{\text{on}}*L+k_{\text{off}},$$

$$\text{Occupancy}=L/\left(L+K_{d}\right),$$

$$Y_{\max}=\text{Occupancy}*B_{\max},$$

$$\text{drift}=B_{\max}*\exp\left(-\text{drift}*X\right),$$

$$Y=\left(Y_{\max}*\left(1-\exp\left(-1*k_{\text{ob}}*X\right)\right)\right)*\text{drift}.$$ (3)

Saturation binding curves for Venus-mini-G_s_ binding to the agonist TS-SNAP-β_2_AR-nLuc were fitted to a one-site specific binding model according to Equation 4. The final *K*_d_ values were taken as an average of *K*_d_ values from individual specific curve fits.

$$Y=\frac{Bmax*X\;}{\left(K_{d}+X\right)},$$ (4)

where Y is the specific binding, *K*_d_ is the equilibrium dissociation constant of the labelled ligand (in this case, Venus-mini-G_s_), and x represents [Venus-mini-G_s_] in nM.

### Statistical analysis

Pearson’s correlation coefficient was used to investigate correlations between mini-G_s_ recruitment, CASE-G_s_ activation, mini-G_s_ binding *K*_d_, *k*_on_ and *k*_off_ values, and literature p*K*_i/d_. Deming regression was applied to determine the line of best fit while accounting for errors in observations on both the x- and y-axes. All statistical analyses were performed in GraphPad Prism 9, and p < 0.05 was considered statistically significant.

## Results

### Characterisation of β_2_AR agonist efficacy for G_s_ activation

**FIGURE 1:**
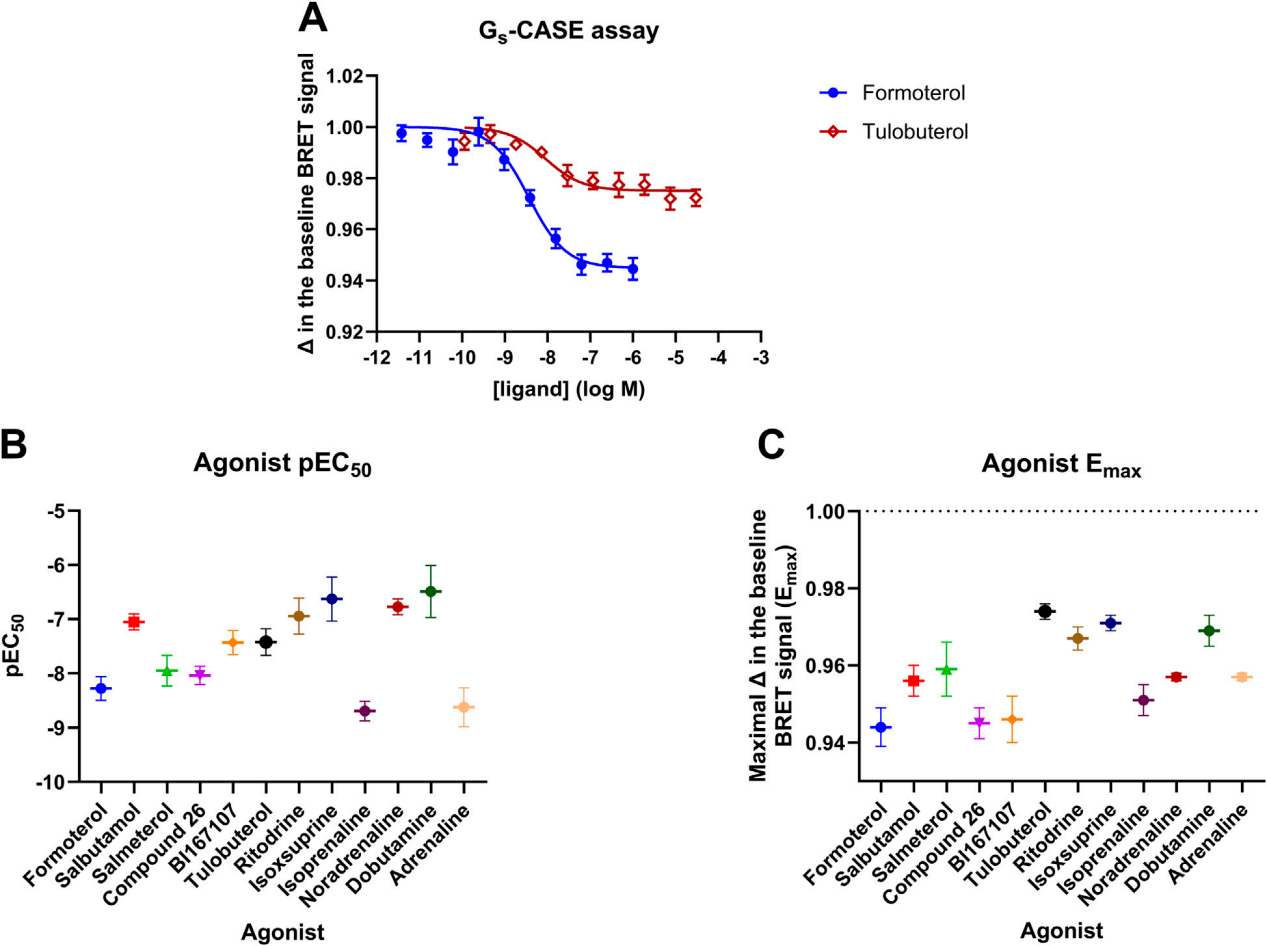
T-REx^TM^-293-SNAP-β_2_AR G_s_-CASE activation assay. **(A)** Concentration-response curves are shown for the full agonist formoterol and the partial agonist tulobuterol. The Gs-CASE baseline BRET signal was set to 1 for normalisation purposes. The response to each agonist is expressed as a fractional change relative to the basal response. Response data are representative of three or more experiments. **(B)** Gs-CASE pEC_50_ and **(C)** E_max_ values are shown for the 12 agonists. Data are presented as the mean ± SEM of three or more experiments.

To produce a suitable dataset for analysis, we chose 12 β_2_AR agonists anticipated to have a diverse range of efficacies, affinities, and ligand binding kinetics. We first characterised the efficacy of these compounds in activating the heterotrimeric G_s_ protein using a NanoBRET-based biosensor (Schihada et al., 2021; Harwood et al., 2024). In this assay format, G_s_ protein activation results in a decrease in the NanoBRET signal as the nLuc-labelled α-subunit of the G_s_ protein dissociates from the Venus-labelled γ-subunit. These experiments are summarised in Figures 1A–C and Table 1.

**TABLE 1:** Summary of efficacy and potency values obtained for β_2_AR agonists in the G_s_-CASE activation assay.

	Gs-CASE assay	
	pEC_50_	E_max_
Formoterol	8.28 ± 0.22	0.944 ± 0.005
Salbutamol	7.05 ± 0.15	0.956 ± 0.004
Salmeterol	7.95 ± 0.28	0.959 ± 0.007
Compound 26	8.03 ± 0.17	0.945 ± 0.004
BI167107	7.43 ± 0.22	0.946 ± 0.006
Tulobuterol	7.42 ± 0.25	9.974 ± 0.002
Ritodrine	6.95 ± 0.33	0.967 ± 0.003
Isoxsuprine	6.63 ± 0.41	0.971 ± 0.002
Isoprenaline	8.69 ± 0.18	0.951 ± 0.004
Noradrenaline	6.77 ± 0.15	0.957 ± 0.001
Dobutamine	6.49 ± 0.48	0.969 ± 0.004
Adrenaline	8.62 ± 0.36	0.957 ± 0.001

The T-REx™-293-SNAP-β_2_AR CASE G_s_ stable cell line was induced with 1 μg/mL tetracycline for 48 h. The Gs-CASE response of each agonist was expressed as a fractional change in the basal response. Values are presented as the mean ± SEM of three or more experiments.

The Gs-CASE assay functions as a non-amplified system, showing very distinct differences in measurable efficacy between full and partial agonists. The concentration-response curves for formoterol (full) and tulobuterol (partial agonist) are shown in Figure 1A. A broad range of potencies was observed for the 12 tested ligands, with pEC_50_ values ranging from 6.49 ± 0.48 for dobutamine to 8.69 ± 0.18 for isoprenaline (see Figure 1B; Table 1). Figure 1C shows a range of efficacy values for each agonist, represented by E_max_ (maximal decrease in basal BRET) values, with the lowest efficacy agonists being tulobuterol and isoxsuprine and the highest being formoterol and Compound 26.

### Validation of mini-G_s_ proteins as tools for probing G_s_ protein binding

**FIGURE 2:**
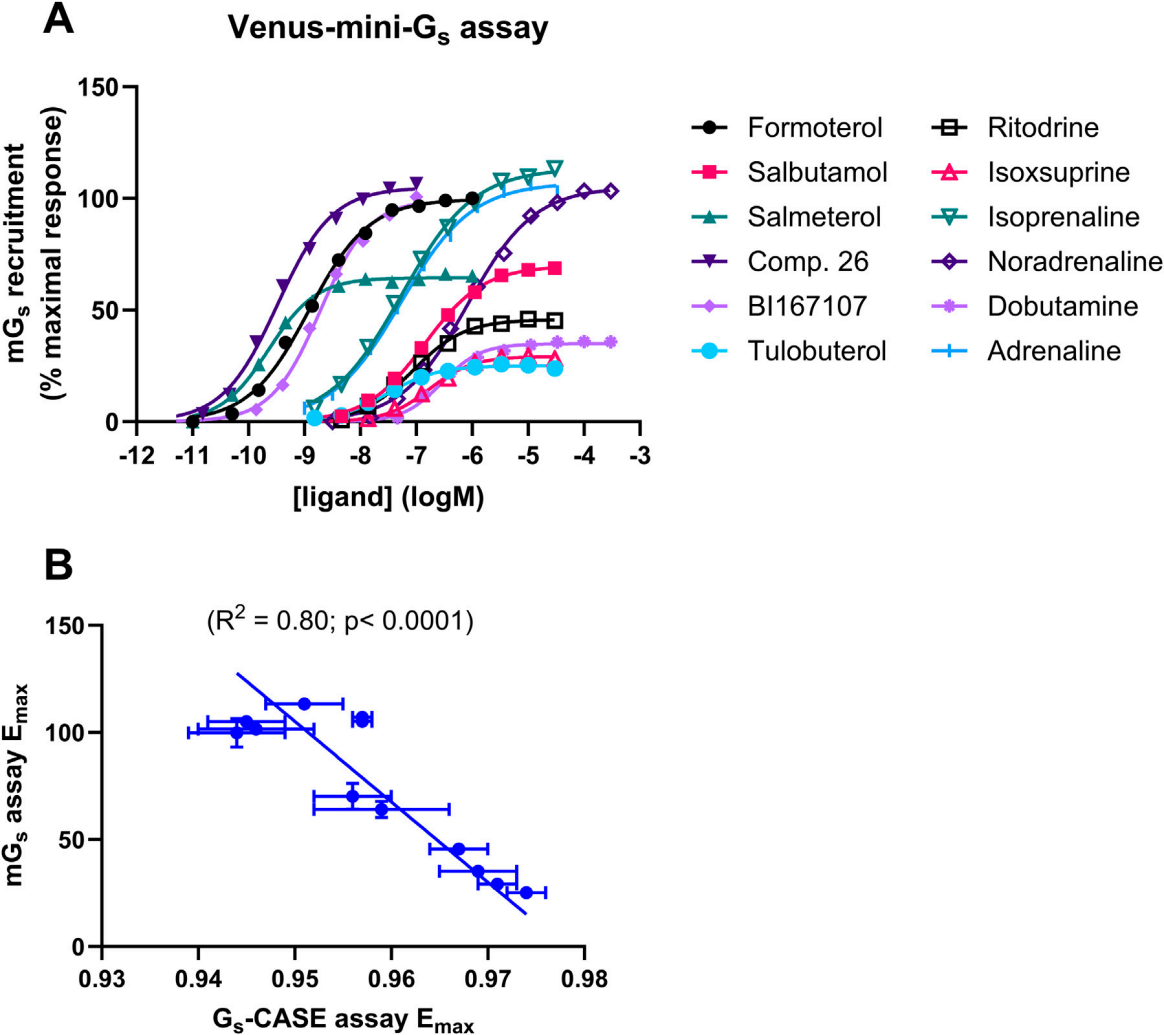
β_2_AR ligand efficacy determined in the Venus-mini-G_s_ recruitment assay. **(A)** Ligand-induced increases in BRET, following the recruitment of Venus-labelled mini-G_s_ to β_2_AR-nLuc. The Venus-mini-G_s_ ligand-response amplitude of each agonist was compared to the maximal response of formoterol (1 μM). **(B)** Correlation of the Gs-CASE assay-response amplitude, expressed as the maximal Δ (E_max_) in the baseline BRET signal, with E_max_ measured in the mini-Gs recruitment assay. Data are presented as the mean ± SEM of three experiments.

In order to investigate the mechanism underlying the differences in efficacy, we expressed and purified fluorescently labelled mini-G_s_ proteins from *E*. coli (Supplementary Figure 1); our aim was to probe the affinity and binding kinetics of Venus-mini-G_s_ protein for the agonist-bound β_2_AR–nLuc complex using NanoBRET. Figure 2A shows that all 12 agonists recruited Venus-mini-G_s_ protein to β_2_AR-nLuc in HEK cell membranes in a concentration-dependent manner, with varying E_max_ and pEC_5o_ values (Table 2). Moreover, Figure 2B reveals a strong correlation (R^2^ = 0.80, p = 0.0001) between E_max_ values for mini-G_s_ recruitment and E_max_ values for G_s_-CASE activation, further validating these assays as effective tools for investigating β_2_AR–G_s_ interactions.

**TABLE 2:** Summary of mini-Gs assay potency (pEC_50_) and efficacy (E_max_) values and literature pK_i_ values for the 12 agonists of varied efficacy under study.

	Mini-Gs recruitment assay		Radioligand binding
	pEC_50_	E_max_ (% formoterol response)	p*K*_i_ or p*K*_d_
Formoterol	8.92 ± 0.09	99.9 ± 6.7	8.63 ± 0.02
Salbutamol	6.85 ± 0.09	70.2 ± 6.0	6.01 ± 0.03
Salmeterol	9.64 ± 0.08	64.1 ± 3.8	9.26 ± 0.06
Compound 26	9.48 ± 0.03	105.1 ± 1.6	*9.81 ± 0.09
BI167107	9.48 ± 0.03	101.6 ± 0.83	**10.1
Tulobuterol	7.50 ± 0.04	25.1 ± 2.3	6.83 ± 0.09
Ritodrine	7.07 ± 0.08	45.6 ± 2.9	5.81 ± 0.07
Isoxsuprine	6.76 ± 0.14	29.2 ± 2.4	5.93 ± 0.09
Isoprenaline	7.27 ± 0.14	113.4 ± 2.0	6.64 ± 0.09
Noradrenaline	6.08 ± 0.06	105.1 ± 1.3	5.41 ± 0.07
Dobutamine	6.52 ± 0.08	35.2 ± 0.9	5.84 ± 0.05
Adrenaline	7.30 ± 0.08	107.0 ± 2.9	6.13 ± 0.05

The mini-Gs assay values are presented as the mean of three experiments ±SEM. Literature binding p*K*_i_/p*K*_d_ values are taken from Baker (2010), Rasmussen et al. (2011a), Rosethorne et al. (2016), Baker (2010), Rosethorne et al. (2016), and Rasmussen et al. (2011b). The Venus-labelled mini-G_s_ ligand-response amplitude of each agonist was compared to the maximal response of formoterol (1 μM). Data are shown as the mean ± SEM of three experiments.

### Investigating the kinetics of mini-G_s_ protein binding to the β_2_AR in complex with agonists of varying efficacies

**FIGURE 3:**
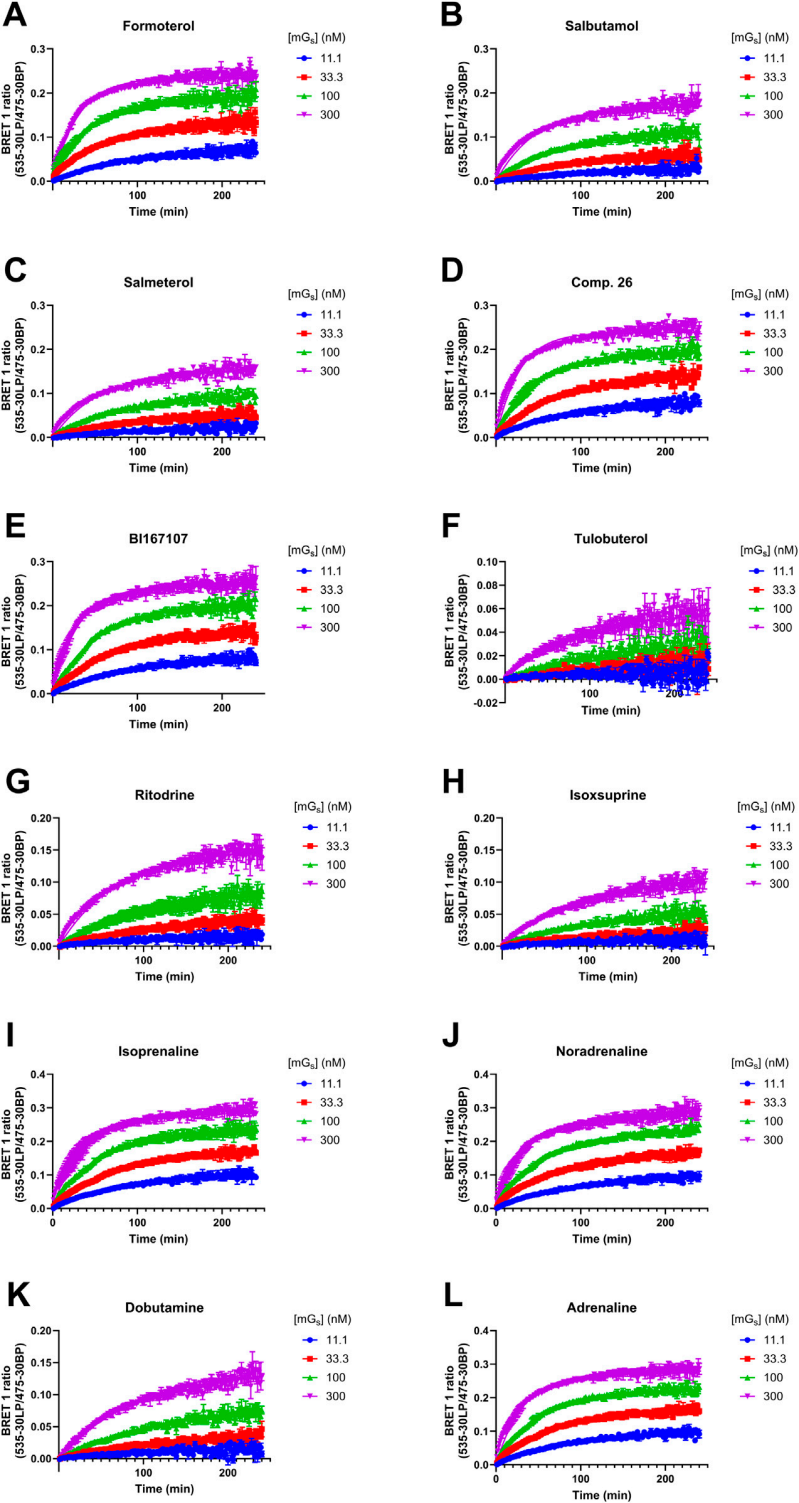
Kinetics of the association of Venus-mini-G_s_ to the agonist β_2_AR–nLuc complex, as measured using nanoBRET. Recruitment of the mini-G_s_ protein by **(A)** formoterol, **(B)** salbutamol, **(C)** salmeterol, **(D)** Compound 26, **(E)** BI167107, **(F)** tulobuterol, **(G)** ritodrine, **(H)** isoxsuprine, **(I)** isoprenaline, **(J)** noradrenaline, **(K)** dobutamine, and **(L)** adrenaline. Data are presented as the mean ± SEM of three experiments.

We established a kinetic NanoBRET binding assay to measure Venus-mini-G_s_ protein recruitment to β_2_AR-nLuc in membrane preparations. To achieve this, we pre-incubated receptor-containing membranes with a saturating concentration (×100 EC_50_) of each β_2_AR agonist, as characterised above. The pre-incubated membranes were then added to a plate containing various concentrations of Venus-mini-G_s_ protein, and we measured the association between these two proteins using NanoBRET (Figure 3; Table 3). Both association and dissociation rates (*k*_on_ and *k*_off_) of Venus-mini-G_s_ for agonist β_2_AR-nLuc could be obtained by analysing the observed association kinetics (Table 3). These studies showed that the full agonists, isoprenaline (*k*_on_ = 3.00 ± 0.1 × 10^5^ M^−1^ min^−1^) and adrenaline (*k*_on_ = 3.06 ± 0.15 × 10^5^ M^−1^ min^−1^), induce faster recruitment of the mini-G_s_ protein than the partial agonists, ritodrine (*k*_on_ = 6.13 ± 0.75 × 10^4^ M^−1^ min^−1^) and isoxsuprine (*k*_on_ = 4.97 ± 0.29 × 10^4^ M^−1^ min^−1^). *k*_off_ values were similar for all ligands, with all values within the range of 0.0070–0.0113 min^−1^. We also conducted these mini-G_s_ kinetics studies on β_2_AR-nLuc extracted into DDM detergent micelles, using 6 of the 12 ligands (Supplementary Figure 2; Supplementary Table 1) and observed similar results.

**TABLE 3:** Summary of mean *k*_off_, *k*_on_, and *K*_d_ values for purified Venus-mini-G_s_ recruitment to TS-SNAP-**β**_2_AR-nLuc by various β_2_AR agonists; NanoBRET between TS-SNAP-β_2_AR-nLuc and Venus-mini-G_s_ read on PHERAstar FSX, at room temperature, using the BRET1 module.

	Mini-G_s_ recruitment assay		
	Mini-G_*s*_ *k*_off_ (min^−1^)	Mini-G_*s*_ *k*_on_ (M^−1^ min^−1^)	Mini-G_*s*_ *K*_d_ (nM)
Formoterol	0.0084 ± 0.0003	2.77 ± 0.09 × 10^5^	30.4 ± 2.0
Salbutamol	0.0113 ± 0.0003	1.16 ± 0.04 × 10^5^	97.5 ± 5.9
Salmeterol	0.0109 ± 0.0007	9.18 ± 1.24 × 10^4^	126 ± 27
Compound 26	0.0076 ± 0.0007	2.73 ± 0.13 × 10^5^	27.8 ± 1.2
BI167107	0.0070 ± 0.0002	2.58 ± 0.07 × 10^5^	27.1 ± 1.3
Tulobuterol	0.0073 ± 0.0015	4.43 ± 0.39 × 10^4^	161 ± 21
Ritodrine	0.0107 ± 0.0004	6.13 ± 0.75 × 10^4^	182 ± 32
Isoxsuprine	0.0096 ± 0.0011	4.97 ± 0.29 × 10^4^	193 ± 11
Isoprenaline	0.0073 ± 0.0003	3.00 ± 0.11 × 10^5^	24.5 ± 1.6
Noradrenaline	0.0076 ± 0.0012	2.99 ± 0.25 × 10^5^	25.0 ± 2.0
Dobutamine	0.0008 ± 0.0007	6.16 ± 0.26 × 10^4^	133 ± 5
Adrenaline	0.0077 ± 0.0006	3.06 ± 0.15 × 10^5^	25.1 ± 0.1

Values are presented as the mean ± SEM of three independent experiments.

**FIGURE 4:**
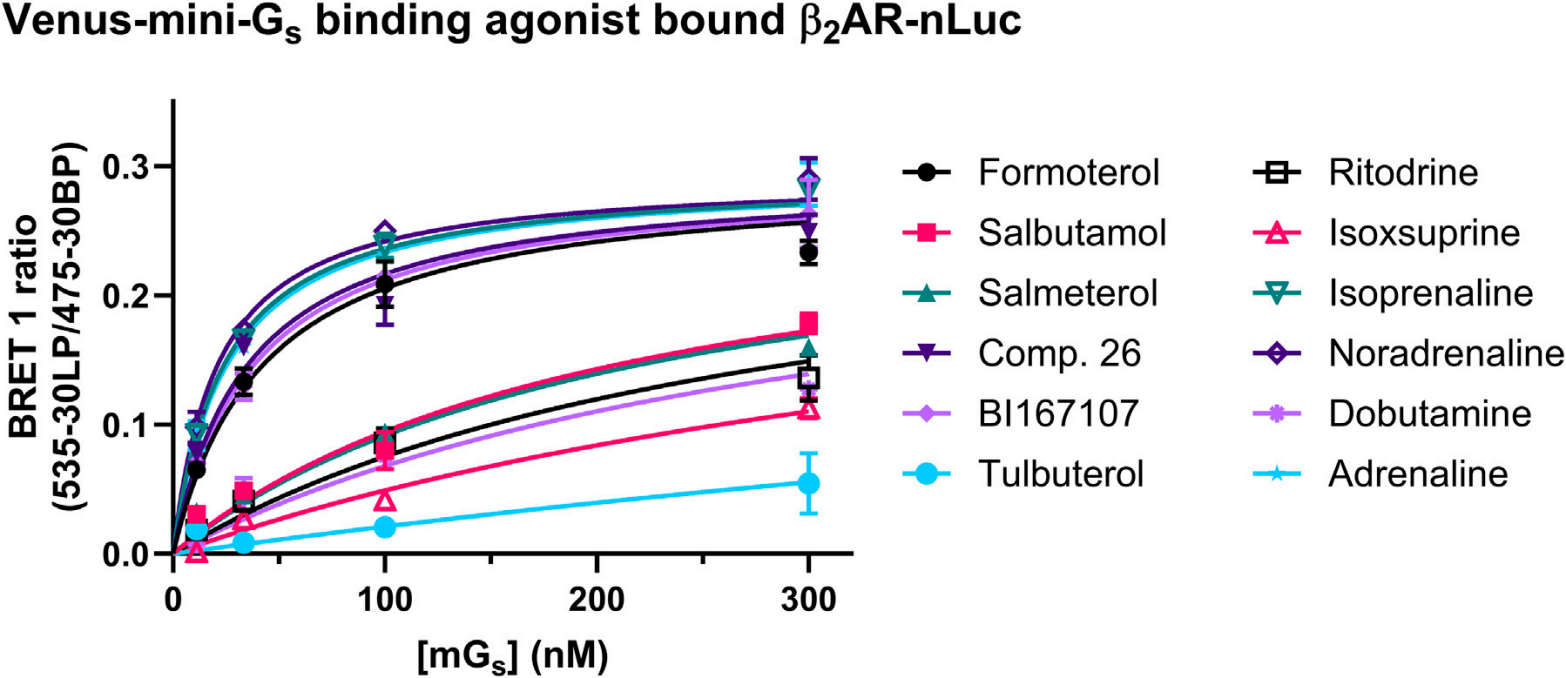
Equilibrium binding of Venus-mini-G_s_ bound to β_2_AR-nLuc at saturating concentrations of each agonist, as reflected by an increase in the BRET signal. The full agonist β_2_AR complexes recruit Venus-mini-G_s_ with higher affinity compared to partial agonist β_2_AR mG complexes. Data shown are presented as the mean ± SEM of three experiments.

To probe the binding affinity of the Venus mini-G_s_ protein to the agonist β_2_AR–nLuc complex, we added ligands in excess (×100 reported pEC_50_ determined in the mini-G_s_ recruitment assay, see above) and incubated with the membrane fraction expressing β_2_AR-nLuc for 15 min prior to the addition of Venus-labelled mini-G_s_ (Figure 4). The resulting affinity (p*K*_d_) values are summarised in Table 3, which ranged from 24 nM for the full agonist isoprenaline to 193 nM for the partial agonist isoxsuprine. These data also showed a difference in the maximum amount of mini-G_s_ protein (E_max_) recruited over the limited concentration range studied (300–10 nM), with full agonists exhibiting higher recruitment compared to partial agonists.

### Affinity and the rate of association of Venus-mini-Gs protein for β_2_AR-nLuc correlated with agonist efficacy

**FIGURE 5:**
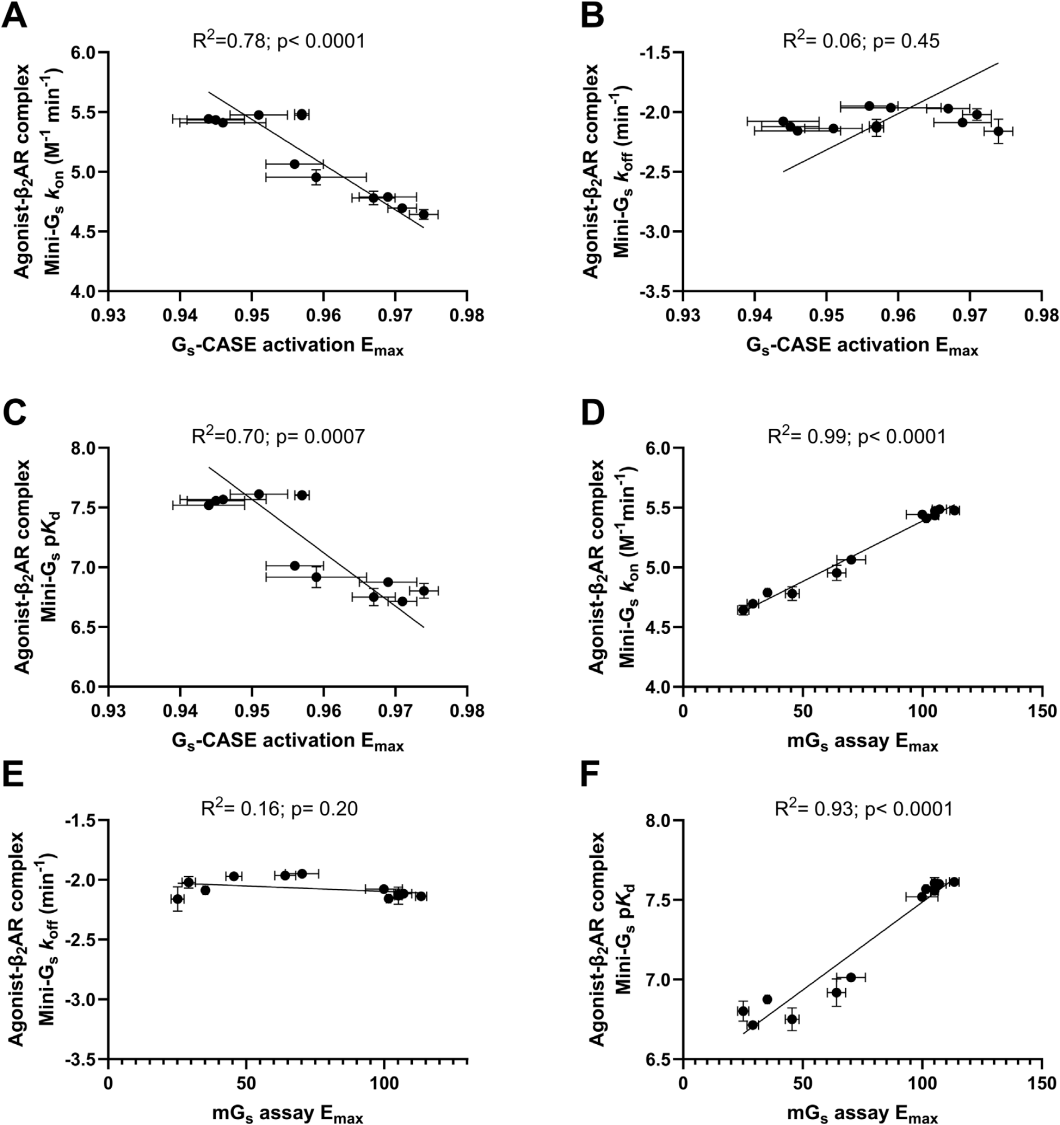
Correlation plots of G_s_-CASE activation E_max_ with **(A)** agonist β_2_AR complex Venus-mini-G_s_ association rate (*k*_on_), **(B)** β_2_AR complex Venus-mini-G_s_ affinity (p*K*_d_), and **(C)** β_2_AR complex Venus-mini-G_s_ dissociation rate (*k*_off_). The GS-CASE response to each agonist is expressed as a fractional change relative to the basal response. Correlation plots of mini-G_s_ assay E_max_ with **(D)** agonist β_2_AR complex Venus-mini-G_s_ association rate (*k*_on_), **(E)** β_2_AR complex Venus-mini-G_s_ affinity (p*K*_d_), and **(F)** β_2_AR complex Venus-mini-G_s_ dissociation rate (*k*_off_). The Venus-labelled mini-G_s_ ligand-response amplitude of each agonist in the mini-G_s_ assay was compared to the maximal response of formoterol (1 μM). Deming regression was applied to determine the line of best fit. Data are shown as the mean ± SEM of three experiments.

Finally, we performed Pearson’s correlation analysis between both the association rates (*k*_on_) and dissociation rates (*k*_off_) and the affinity (pK_d_) values for Venus-mini-Gs binding agonist β_2_AR–nLuc complexes vs. agonist efficacy, comparing both G_s_-CASE and mini-G_s_ assay E_max_ values (Figure 5). This analysis showed a strong correlation between ligand efficacy (E_max_) measured in both assay formats and mini-G_s_ association rates (*k*_on_) (R^2^ = 0.78, p < 0.0001 and R^2^= 0.99, p < 0.0001 respectively; see Figures 5A, D) and between ligand efficacy (E_max_) and mini-G_s_ affinity (pK_d_) (R^2^ 0.70, p = 0.0007 and R^2^ = 0.93, p < 0.0001, respectively; see Figures 5C, F). This suggests that the differences in agonist efficacy can be explained by agonist β_2_AR complexes’ ability to recruit the G_s_ protein. No correlation was observed between ligand efficacy (E_max_) measured in either assay formats and mini-G_s_ dissociation rates (*k*_off_) (R^2^ = 0.06, p = 0.45 and R^2^ = 0.16, p = 0.20, respectively; see Figures 5B, E).

We also performed this same correlation analysis between these mini-G_s_ kinetics values obtained in detergent micelles and G_s_ efficacy data obtained in the Gs-CASE assay and found a similar trend (Supplementary Figure 4).

### Efficacy of β_2_AR agonists does not correlate with ligand binding kinetics

Previous studies have suggested that for some GPCRs, there is a relationship between ligand efficacy and the dissociation rates of ligand binding (Sykes et al., 2009a; Guo et al., 2016). To investigate the correlations between ligand residence time and efficacy, we analyzed existing kinetic data. This analysis revealed a broad range of measured *k*_off_ values, with adrenaline exhibiting the fastest dissociation rate and Compound 26 showing the slowest.

**FIGURE 6:**
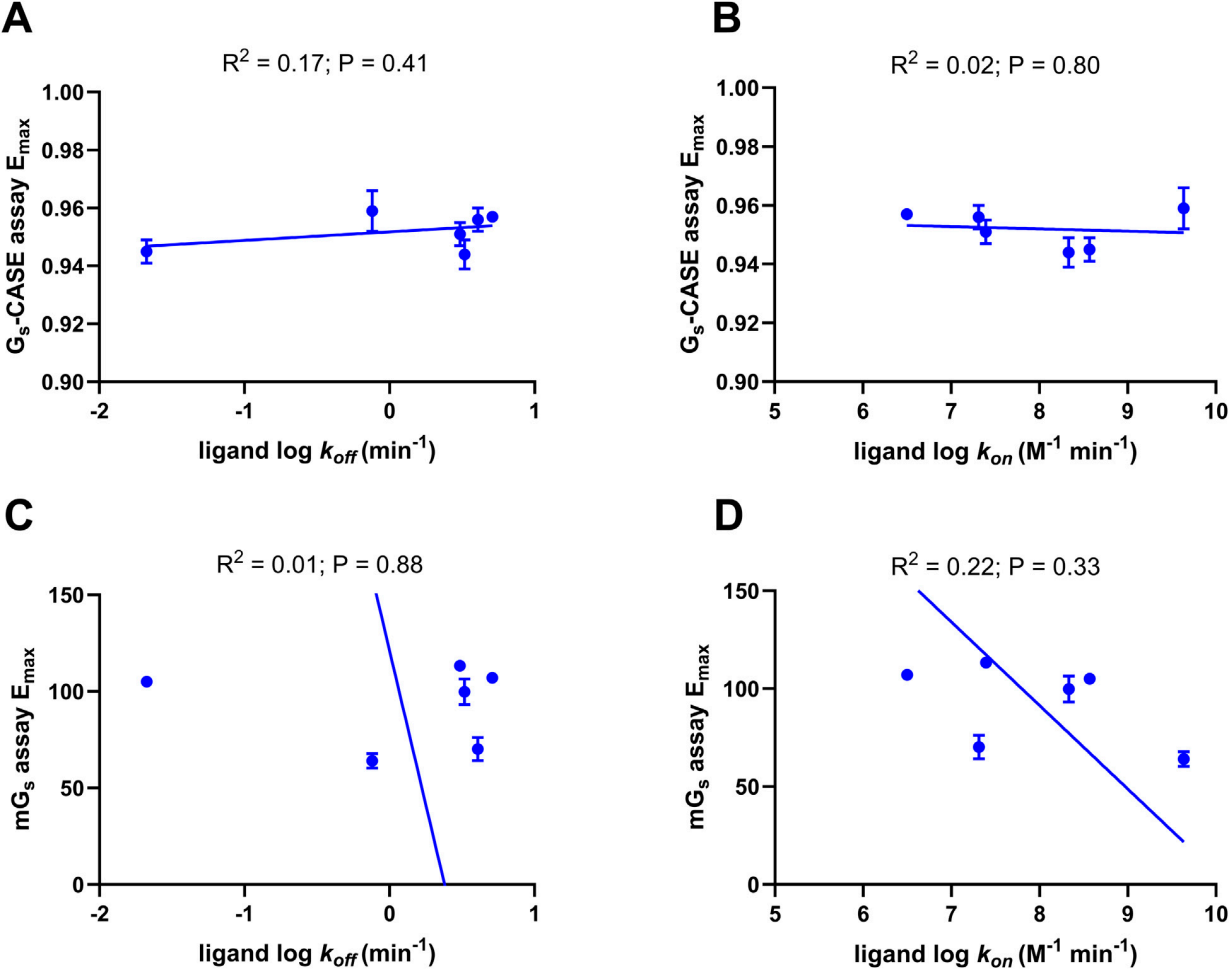
Correlation plots of ligand efficacy with ligand binding parameters. Plot of **(A)** ligand dissociation rates (*k*_off_) with G_s_-CASE activation E_max_ and **(B)** ligand association rate (*k*_on_) with G_s_-CASE activation E_max_. The Gs-CASE assay response amplitude is expressed as the maximal Δ (E_max_) in the baseline BRET signal, which was set to 1.0. Plot of **(C)** ligand dissociation rates (*k*_off_) with mini-G_s_ assay E_max_ and **(D)** ligand association rate (*k*_on_) with mini-G_s_ assay E_max_. The Venus-labelled mini-G_s_ ligand-response amplitude of each agonist in the mini-G_s_ assay was compared to the maximal response of formoterol (1 μM). Deming regression was applied to determine the line of best fit. Data are shown as the mean ± SEM of three experiments. Ligand association and dissociation rates were taken from Sykes et al., 2014, Sykes et al., 2012 and Rosethorne et al., 2016.

The relationships between agonist efficacy, as determined by E_max_ values obtained from the Gs-CASE and mini-G_s_ recruitment assays, and literature ligand binding association (*k*_on_) and dissociation rates (*k*_off_) were determined using Pearson’s correlation analysis (see Figure 6). This analysis showed no statistically significant correlation between ligand *k*_off_ values and the efficacy values determined for 6 of the 12 β_2_AR agonists. Moreover, we also conducted kinetic TR-FRET-based ligand binding studies on 6 of the 12 β_2_AR agonists in detergent micelles (Supplementary Figure 5) and found no statistically significant correlation (R^2^ = 0.26, p = 0.29) between relative ligand residence times (IC_50_ 1 min/IC_50_ equilibrium) and their efficacy (Supplementary Figure 6; Supplementary Table 2).

## Discussion

In this study, we aimed to investigate the molecular basis for ligand efficacy. The first hypothesis was that the ligand binding kinetics, or ligand residence time, may influence efficacy. The second hypothesis was that the kinetics of G protein recruitment to the receptor–agonist complex may be correlated to ligand efficacy.

We found no correlations between the ligand binding kinetics and its efficacy. Whilst some studies suggested a role for ligand dissociation kinetics (Guo et al., 2012; Sykes et al., 2009) for adenosine A2_A_ and muscarinic M_3_ receptors, our data are congruent with the previously reported observation that it was not the case for β_2_AR (Sykes and Charlton, 2012).

Alternatively, we observed a linear correlation between ligand-induced differences in mini-G_s_ protein binding kinetics (*k*_on_) and affinity (p*K*_d_) for the agonist-bound β_2_AR and agonist efficacy, the ability of a ligand to activate the heterotrimeric G_s_ protein. In contrast, our data showed minimal difference in the dissociation rate (*k*_off)_ or corresponding residence time (1/*k*_off_) of the Venus-mini-G_s_ when binding to different agonist-β_2_AR complexes. Since the affinity of mini-G_s_ is a ratio of *k*_on_ and *k*_off_ and mini-G_s_ *k*_off_ appears relatively constant, it is expected that mini-G_s_ *k*_on_ and its affinity correlate.

**FIGURE 7:**
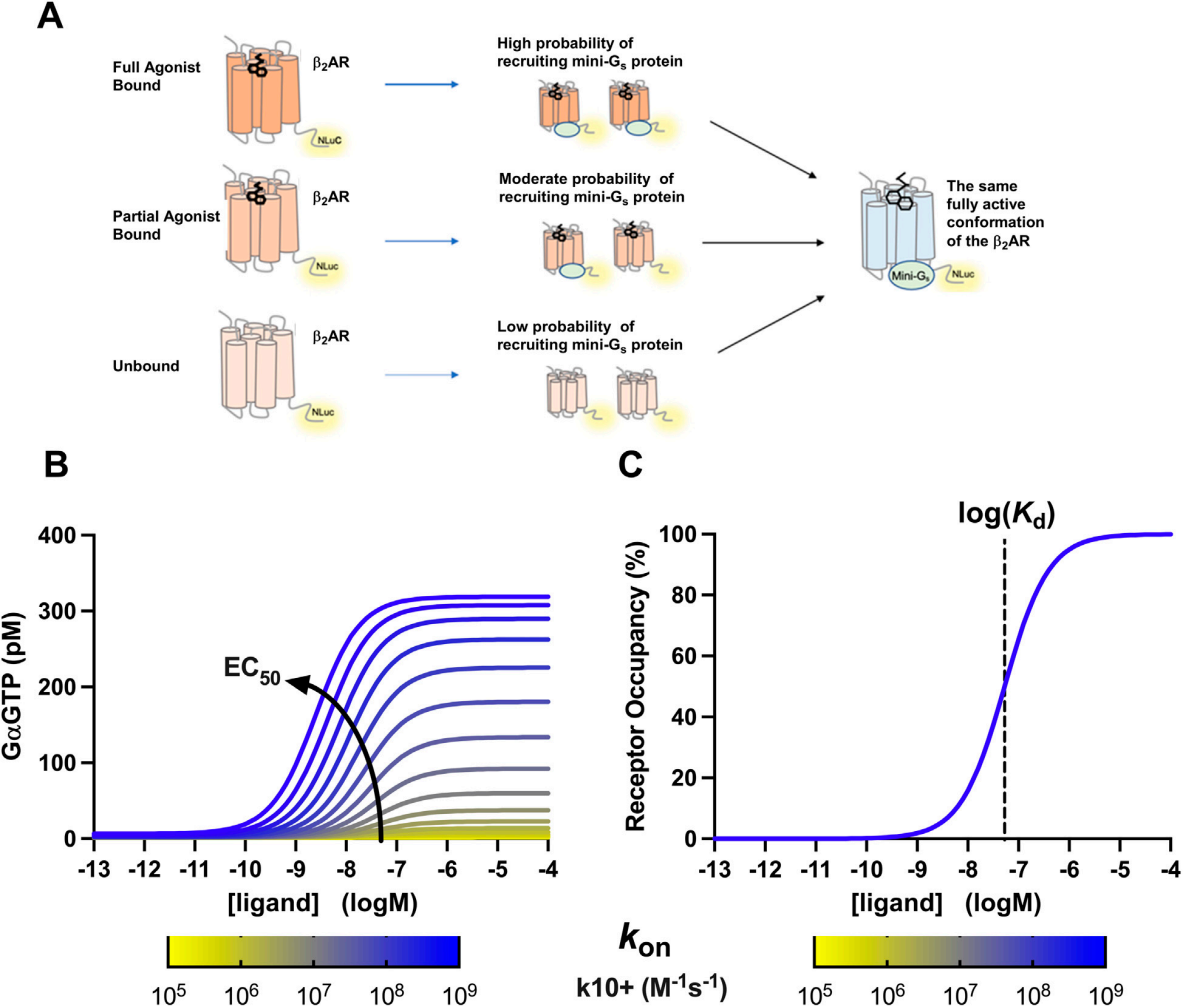
Conformational model of efficacy proposed by this study: **(A)** agonists of higher efficacy induce a conformation of β_2_AR that is more likely to recruit a mini-G_s_ protein, but once bound, there is no difference in the β_2_AR conformation within the agonist β_2_AR-mini–G_s_ complex. **(B)** Use of the cubic ternary complex model to investigate the effect of increasing the rate of G protein recruitment on the potency of the agonist–receptor complex to activate the G protein. Arrow indicates increases in apparent ligand EC_50_ values for the formation of GαGTP. **(C)** Use of the cubic ternary complex model to investigate the effect of increasing the rate of G protein recruitment on agonist affinity for the GPCR. Dotted line indicates log (*K*_d_) of ligand-receptor occupancy. As shown in the figure, the association rate of Gα to the receptor does not affect ligand binding affinity; hence, the yellow and blue curves lie directly on top of each other.

Our hypothesis is that agonist binding to the β_2_AR increases the propensity for G protein recruitment, which underlies the molecular basis of ligand efficacy at the β_2_AR (Figure 7A). To support our hypothesis, we applied a previously validated mathematical model of the cubic ternary complex model (BioModels ID:2306220001) to investigate the effect of increasing the forward rate of G protein binding to the activated receptor, on both G protein activation and agonist–receptor occupancy at the β_2_AR (Figures 7B, C). As indicated, increasing the on-rate for G protein recruitment increases the efficacy and potency of G protein activation by the ligand, without changing agonist–receptor occupancy (Figure 7B). This, therefore, supports our hypothesis that an increase in G protein recruitment propensity underlies the molecular basis of ligand efficacy at the β_2_AR.

These differences in the rate of mini-G_s_ recruitment and the resulting differences in mini-G_s_ affinity suggest that subtle differences in agonist β_2_AR complex conformations result in differences in agonist efficacy due of differences in the ability of these conformations to affect the recruitment of Venus-mini-G_s_. As the dissociation rates of the mini-G_s_ protein are very similar for all ligands, the structure of the GPCR–G protein complex is likely similar for all ligands. This hypothesis aligns with recent observations made by NMR (Jones et al., 2024), where the full agonist isoprenaline induced a different conformational state of the β_1_ adrenergic receptor (β_1_AR) compared to the partial agonists xamoterol and salbutamol. However, the conformations were similar in the case of the ternary complex with mini-G_s_. The authors also reported faster recruitment kinetics for the full agonist isoprenaline, a result that aligns well with our own observations for a wide range of partial and full agonists, as presented in this study.

Moreover, this conformational model (see Figure 7) is supported by data from hydrogen/deuterium exchange mass spectrometry (HDMS) and hydroxy radical foot printing mass spectrometry (HDX) (Du et al., 2019), where the conformational changes involved in β_2_AR–G_s_ protein complex formation were investigated. Du et al. showed that the conformation of the initial β_2_AR–G_s_ structure differs from that of the fully formed nucleotide free β_2_AR–G_s_ complex. Furthermore, NMR studies (Nygaard et al., 2013; Manglik et al., 2015) show that the agonist BI-167-107 alone is not sufficient to fully stabilise the β_2_AR in the active state and that nanobody 80 is required to fully stabilise the active state. These data support our findings that the conformation of the agonist β_2_AR complex differs from that of the agonist β_2_AR–mini-G_s_ complex. Moreover, Liu et al. (2012) investigated the conformational states of β_2_AR bound to agonists of a range of efficacies and showed efficacy-dependent differences in the agonist β_2_AR conformational state. Structural studies of the agonist-bound β_2_AR or other class A GPCRs have only been possible in the presence of a G protein mimetics (Rasmussen et al., 2011b) and show only very small conformational differences that do not seem to explain differences in efficacy (Katritch et al., 2009). This further supports our finding that there was no difference in the agonist β_2_AR–mini-G_s_ complex conformation.

We performed the majority of this study in membranes as we believe this environment is the most physiologically relevant for performing a kinetic analysis of ligand-induced mini-G_s_ binding. We also reproduced most of the experiments with receptor isolated in DDM detergent micelles (Supplementary Figures 2–6); this approach gives us confidence that our conclusions are relevant purely at the biophysical level, independent of the regulatory elements of the cell, while also establishing a baseline for future biophysical studies. However, the full applicability of our findings to the native cell environment remains to be fully elucidated. Interestingly, Sungkaworn et al. (2017) investigated the association rate (*k*_on)_ and dissociation rate (*k*_off)_ of Gα_I_ binding to the α2AR receptor in CHO cells in response to a range of agonists using single molecule microscopy. They showed that efficacy is at least partially correlated with *k*_on_ but not *k*_off_ of the Gα_I_ protein. Taken together with the evidence from the current study, this suggests that the conformational model of efficacy proposed may extend to the cellular environment. Future work will investigate whether this model of efficacy proposed is relevant to the β_2_AR in its native cellular environment and whether this model can be generalized as a mechanism for agonist efficacy at other GPCRs.

## Conclusion

In summary, these findings suggest that differences in initial agonist-GPCR conformations, where full agonists stabilise a state that readily recruits G protein, could be central to understanding the molecular basis of efficacy for the 12 β_2_AR agonists studied. In contrast, we found no evidence linking ligand or G protein binding dissociation kinetics to the molecular basis of ligand efficacy at the β_2_AR. We propose a conformational model of efficacy, in which agonists with higher efficacy stabilise a conformation of β_2_AR that is more likely to recruit the G protein. The results from mini-G protein association experiments with ligand prebound to the receptor provide a convenient and direct measurement of ligand efficacy. Further studies incorporating a broader range of agonists with varying efficacies, along with measurements across different receptor types, would help determine whether this mechanism is a general feature of GPCR efficacy.

## Data Availability

The raw data supporting the conclusions of this article will be made available by the authors, without undue reservation.
