# Supplementary material for: Agonist efficacy at the β2AR is driven by the faster association rate of the Gs protein
Source: Front Pharmacol. 2025 Mar 27;16:1367991. doi: 10.3389/fphar.2025.1367991 (PMC11983327; doi:10.3389/fphar.2025.1367991)
Supplement: Supplementary file 1 [file DataSheet1.pdf]

Agonist efficacy at the β_2AR is driven by a faster association rate of the G_s protein.

Clare R. Harwood[1,2], David A. Sykes[1,2,3,*], Theo Redfern-Nichols[4], Owen Underwood[3], Colin Nicholson[1,2], Armin N. Khoshgrudi[1,2], Eline J. Koers[1,2], Graham Ladds[2,4], Stephen J. Briddon[1,2,*] and Dmitry B. Veprintsev [1,2,3,*]

[1]Division of Physiology, Pharmacology and Neuroscience, School of Life Sciences, University of Nottingham, Nottingham NG7 2RD, UK
[2]Centre of Membrane Proteins and Receptors (COMPARE), University of Birmingham and University of Nottingham, Midlands, UK
[3]Z7 Biotech Ltd, Nottingham, UK.
[4]Department of Pharmacology, University of Cambridge, Cambridge CB2 1PD, UK

Correspondence:
David A. Sykes, Stephen J. Briddon and Dmitry B. Veprintsev
david.sykes@nottingham.ac.uk, stephen.briddon@nottingham.ac.uk,
dmitry.veprintsev@nottingham.ac.uk

Supplementary Material

Supplementary Data

Supplementary Figure 1: Production of mini-Gs proteins from *E*. coli: SDS-PAGE gel stained for protein with InstantBlue showing production and purity of His10-Halo-mini-G_s (lanes 1-2), His10-Venus-mini-G_s (lane 3) and His10-mini-G_s proteins (lane 4). Representative gel of n=4 protein preps for His10-Halo-mini-Gs and His10-mini-Gs proteins and n=1 for His10-Venus- mini-G_s.

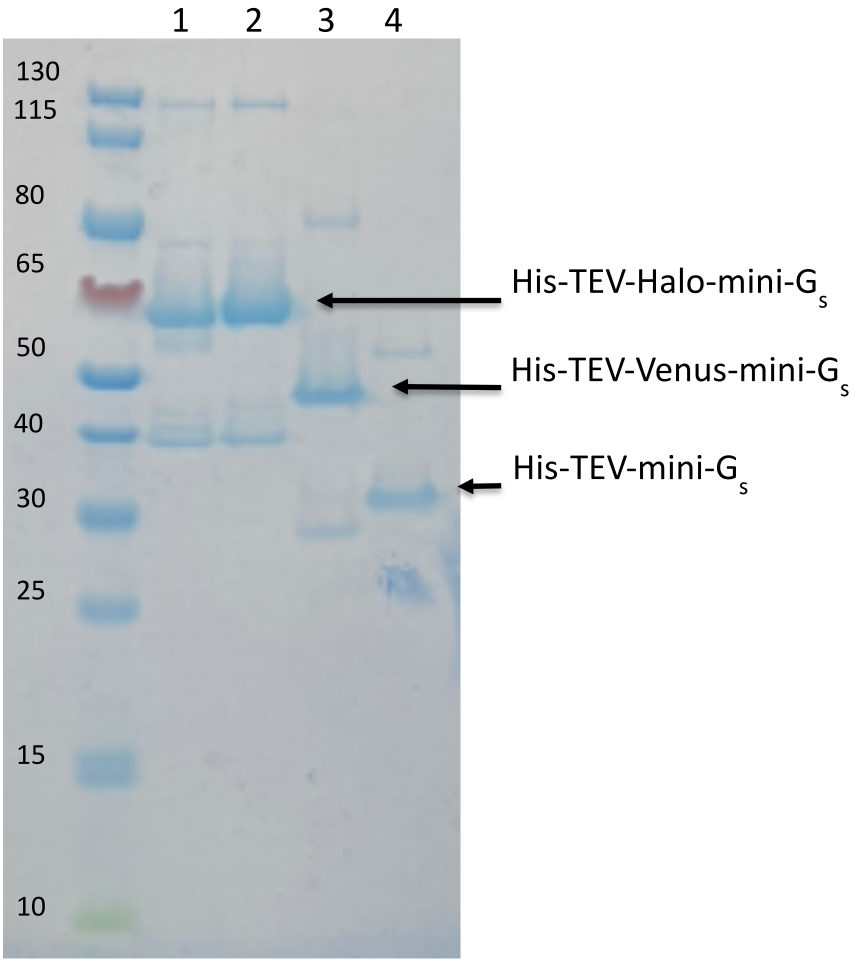

frontiers

Supplementary Figure 2: (A) Investigation of the association and dissociation at 20min using 33µM mini-G_s, of Venus mini-G_s binding to DDM- TS-SNAP-β_2AR when preincubated with saturating concentration of **(B)** Formoterol **(C)** Salmeterol **(D)** BI-167-107 **(E)** Isoprenaline **(F)** adrenaline **(G)** Noradrenaline **(H)** Salbutamol at room temperature, using LUM 550LP/450-80nm module. All figures show specific binding, where 30µM mini-G_s was used to define the NSB, representative raw data of n=3, fitted to a two-phase association and one phase dissociation.

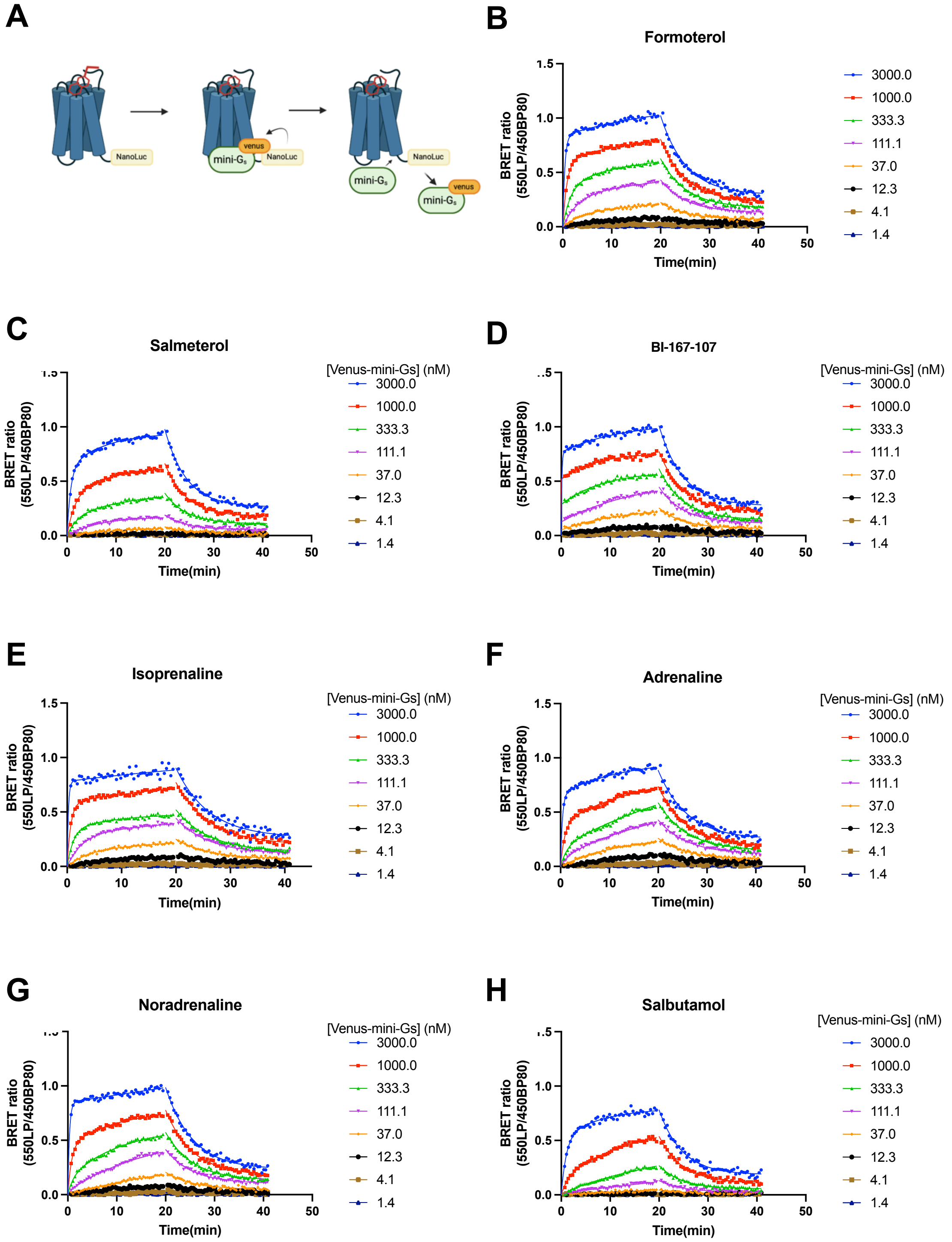

Supplementary Table 1: A summary of the mean pK_d, k_{off}, $k_{on(fast)}$ and residence time values for Venus-mini-G_s proteins binding to the DDM solubilised TS-SNAP-β_2AR bound to the β_2AR agonists BI-167107, formoterol, isoprenaline, adrenaline, noradrenaline, salbutamol and salmeterol, as measured by nanoBRET, values show mean of n=3-4 experiments ± SEM. $^{\%}$denotes full agonist and $^{\$}$partial agonist, as defined by CASE G_s activation assay.

	**pK_d**	**K_{off} (min^{-1})**	**$K_{on(fast)}$ (M^{-1} min^{-1})**	**Residence time (Min)**
$^{\%}$BI-167107	6.7 ±0.03	0.21 ±0.003	7.29 ± 2.16 x10^5	4.76
$^{\%}$Formoterol	6.7 ±0.05	0.20 ±0.011	4.59± 1.64 x10^5	5.00
$^{\%}$Isoprenaline	6.8 ±0.07	0.17 ±0.004	9.19± 0.42 x10^5	4.76
$^{\%}$Adrenaline	6.8 ±0.05	0.18 ±0.014	8.56± 0.13 x10^5	5.50
$^{\%}$Noradrenaline	6.6 ±0.08	0.19 ±0.007	7.92 ±0.56 x10^5	5.20
$^{\$}$Salbutamol	6.0 ±0.07	0.20 ±0.006	3.36±0.64 x10^5	5.00
$^{\$}$Salmeterol	6.1 ±0.09	0.21 ±0.006	4.18±1.2 x10^5	4.76

Supplementary Figure 3: Specific saturation binding of increasing concentrations of purified Venus-mini-G_s binding to DDM-TS-SNAP- β_2AR-nLuc in the presence of saturating concentrations of formoterol, salbutamol, salmeterol BI-167-107, isoprenaline, adrenaline and noradrenaline. nanoBRET between TS-SNAP- β_2AR-nLuc and Venus-mini-G_s was read on PHERAstar FSX, at room temperature, using LUM 550LP/450-80nm module at 20min. Data was fitted to one-site specific binding model, points show the mean ± SEM of 4 independent experiments.

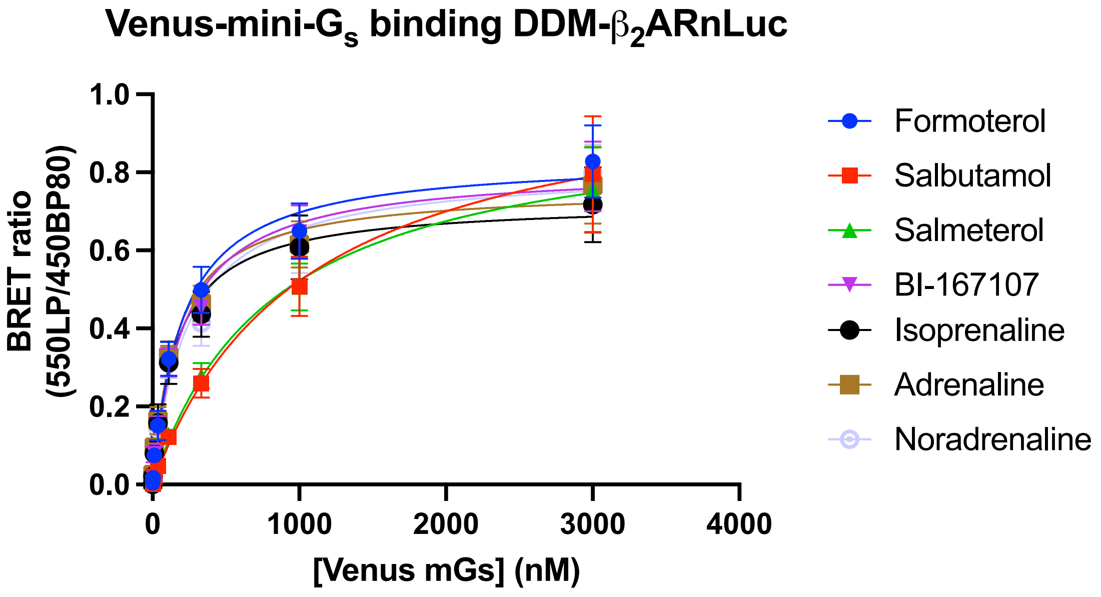

Supplementary Figure 4: Correlation of CASE G_s activation efficacy with **(A)** mini-G_s pK_d and **(B)** mini-G_s k_{on}(fast), (**Supplementary Figure 2 - 3**) for mini-G_s binding DDM-TS-SNAP-β_2AR-nLuc t values were obtained from fitting the operational model on individual data sets with fixed K_A values, correlations were made using Pearson's correlation coefficient, on replicates from n=3-4 experiments for pK_d, k_{on}(fast) and t values.

A

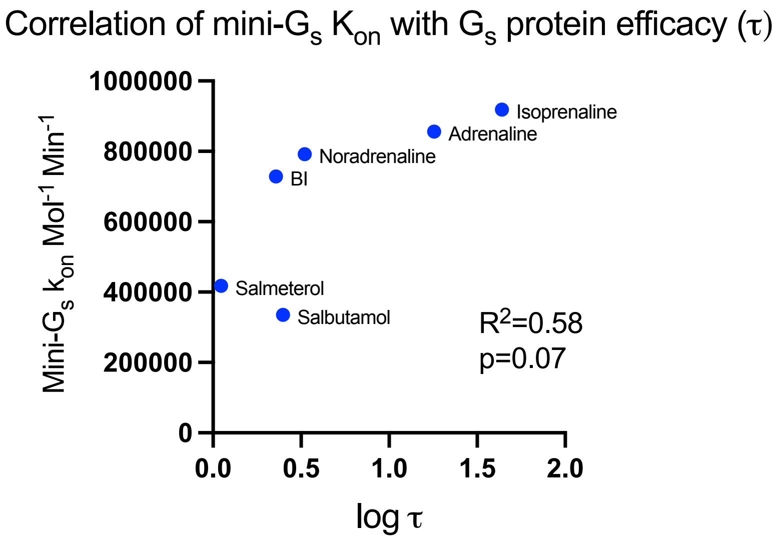

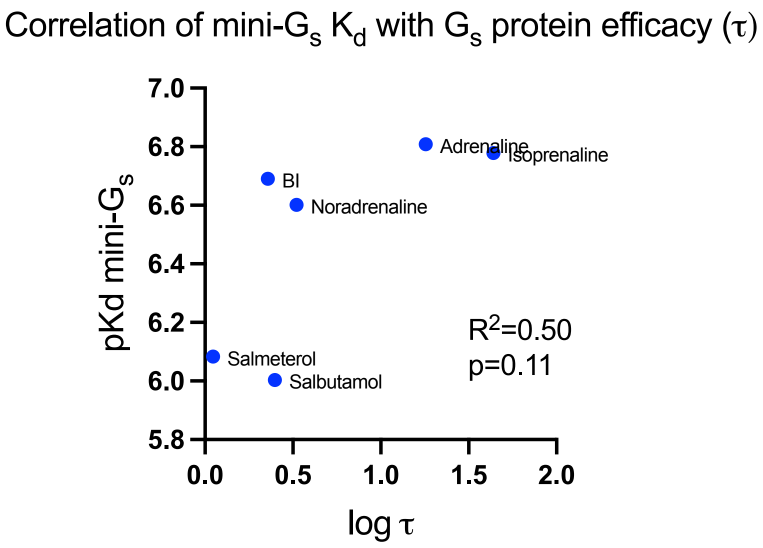

Supplementary Figure 5: Competition binding studies for isoprenaline, adrenaline, noradrenaline, formoterol, salbutamol, salmeterol, BI-167-107 binding Lumi4-Tb labelled DDM-TS-SNAP-b2AR, using 75nM CA200693 (S)-propranolol-green. **(A)** Equilibrium measurements were read at 20 min post DDM-TS SNAP-β_2AR addition for all compounds except BI-167-107 which was read at 40 min. TR-FRET between Lumi4-Tb and CA200693 (S)-propranolol-green PHERAstar FSX using two laser flashes per cycle and 520/620 TRF module. Data points show mean of three experiments normalised to 0% inhibition of specific CA200693 (S)-propranolol-green bound for each compound, ± SEM. **(B)** Comparison of IC_{50} 1min / IC_{50} equilibrium values for all seven compounds binding DDM-TS-SNAP-β_2AR using TR-FRET, bars show mean of three experiments ± SEM.

A

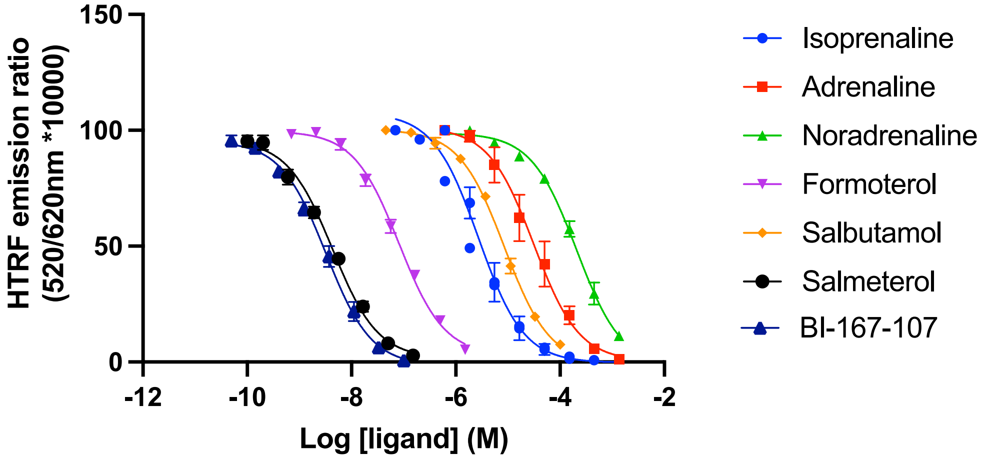

B

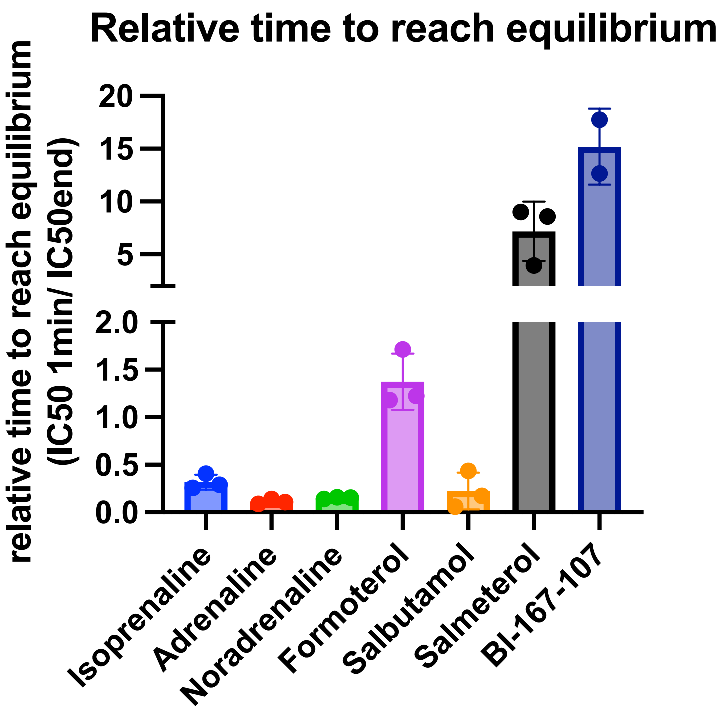

Supplementary Table 2: A summary of pK_i and $IC_{50\ 1min}$ / $IC_{50\ equilibrium}$ values for isoprenaline, adrenaline, noradrenaline, formoterol, salbutamol, salmeterol, and BI-167-107 binding DDM-TS-SNAP-β$_2$AR obtained from equilibrium competition binding. TR-FRET between Lumi4-Tb and CA200693 (S)-propranolol-green PHERAstar FSX using 2 laser flashes per cycle and 520/620 TRF module. Values are mean of three experiments ± SEM.

	**pK_i ± SEM**	**$IC_{50\ 1min}$ /$IC_{50\ equilibrium}$ ± SEM**
Isoprenaline	6.4 ±0.12	0.32 ± 0.05
Adrenaline	5.2 ±0.25	0.11 ±0.01
Noradrenaline	4.4 ±0.09	0.15 ±0.01
Formoterol	7.8 ±0.07	1.37 ±0.17
Salbutamol	5.8 ±0.06	0.22 ±0.11
Salmeterol	9.1 ±0.02	7.2 ±1.63
BI-167-107	9.2 ±0.08	15.2 ±2.08

Supplementary Figure 6: Pearsons' correlation between ligand efficacy (log τ) values and the relative time for these ligands to reach equilibrium (IC_{50} 1min / IC_{50} equilibrium values) (**Supplementary Figure 5**), data points show the mean values calculated from 3 independent experiments.

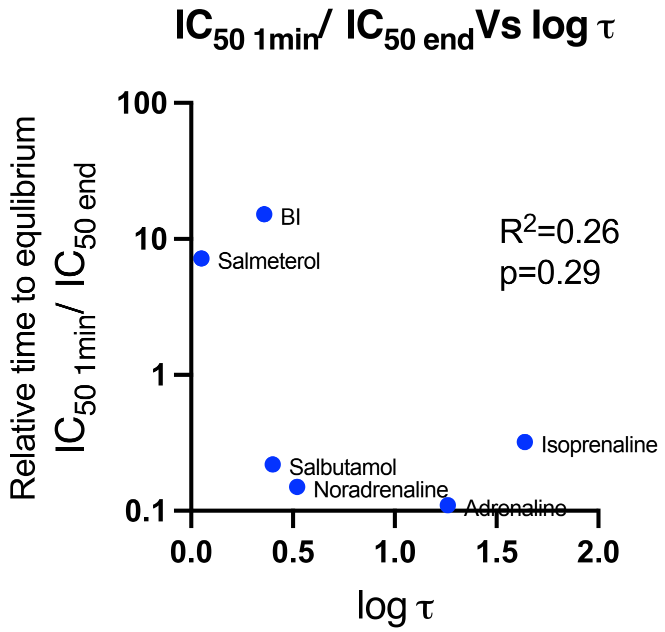

Supplementary Figure 7: Schematic of cubic ternary complex model. A cubic ternary complex model with extended reactions enabling simulation of Gα activation cycle (Biomodels ID:2306220001). Base parameters are shown in **Supplementary Table 3**. To simulate changes in rates of G protein recruitment to active receptor the term β^{+} was varied over a range of 10^{-2} to 10^{4}. Reactions varied by changing the β^{+} are shown in blue.

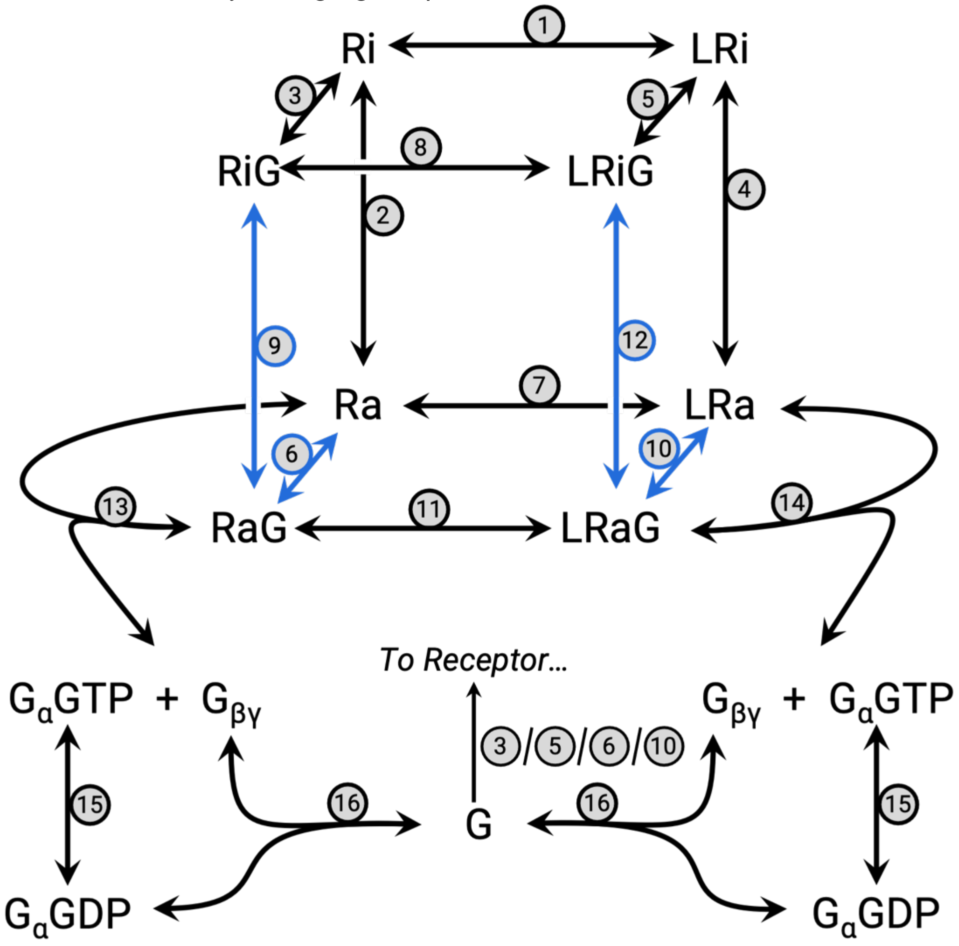

Supplementary Table 3: Base parameter sets for computational simulations. Initial values and descriptions for each reaction parameter and starting species.

Label	Description	Value	Units
k_{L+}	Ligand binding rate	1.00E+05	$M^{-1}\ s^{-1}$
k_{L-}	Ligand unbinding rate	1.00E-02	s^{-1}
k_{act+}	Receptor activation rate	3.00E-02	s^{-1}
k_{act-}	Receptor deactivation rate	1.00E+02	s^{-1}
k_{G+}	G protein binding rate	1.00E+05	$M^{-1}\ s^{-1}$
k_{G-}	G protein unbinding rate	1.10E-02	s^{-1}
α_+	Forward cooperativity factor for ligand bound receptor activation	3.00E+03	-
α_-	Backwards cooperativity factor for ligand bound receptor activation	1.00E-01	-
β_+	Forward cooperativity factor for G protein-bound receptor activation	1.00E+01	-
β_-	Backwards cooperativity factor for G protein-bound receptor activation	1.00E+00	-
γ_+	Forward cooperativity factor for ligand binding a G protein-bound receptor	1.00E+00	-
γ_-	Backwards cooperativity factor for ligand binding a G protein-bound receptor	1.00E+00	-
δ_+	Forward cooperativity factor for ligand bound, G protein-bound receptor activation	1.00E+00	-
δ_-	Backwards cooperativity factor for ligand bound, G protein-bound receptor activation	1.00E+00	-
k_{GDA+}	G protein dissociation rate from active, G protein-bound receptor (possibly ligand-bound)	1.00E+06	s^{-1}
k_{GDA-}	Reformation of active, G protein-bound receptor (possibly ligand-bound)	1.00E-10	$M^{-2}\ s^{-1}$
k_{GRA+}	Heterotrimeric G protein reassociation rate	1.00E+08	$M^{-1}\ s^{-1}$
k_{GRA-}	G protein spontaneous dissociation rate	1.00E-10	s^{-1}
k_{hyd+}	Rate of hydrolysis of GαGTP	1.00E-02	s^{-1}
k_{hyd-}	Spontaneous exchange rate of GDP for GTP	2.00E-06	s^{-1}

**$[Ri]_{initial}$**	Initial concentration of inactive Receptor	4.15E-10	M
**$[G]_{initial}$**	Initial concentration of heterotrimeric G protein	4.15E-10	M
**$[L]_{tot}$**	Total concentration of Ligand	Varies	M
